# Data for evaluation of fast kurtosis strategies, b-value optimization and exploration of diffusion MRI contrast

**DOI:** 10.1038/sdata.2016.72

**Published:** 2016-08-30

**Authors:** Brian Hansen, Sune Nørhøj Jespersen

**Affiliations:** 1 Center of Functionally Integrative Neuroscience (CFIN), Clinical Institute, Aarhus University, 8000 Aarhus C, Denmark; 2 Department of Physics and Astronomy, Aarhus University, 8000 Aarhus C, Denmark

**Keywords:** Medical research, Biomedical engineering, Brain imaging, Magnetic resonance imaging

## Abstract

Here we describe and provide diffusion magnetic resonance imaging (dMRI) data that was acquired in neural tissue and a physical phantom. Data acquired in biological tissue includes: fixed rat brain (acquired at 9.4 T) and spinal cord (acquired at 16.4 T) and in normal human brain (acquired at 3 T). This data was recently used for evaluation of diffusion kurtosis imaging (DKI) contrasts and for comparison to diffusion tensor imaging (DTI) parameter contrast. The data has also been used to optimize b-values for ex vivo and *in vivo* fast kurtosis imaging. The remaining data was obtained in a physical phantom with three orthogonal fiber orientations (fresh asparagus stems) for exploration of the kurtosis fractional anisotropy. However, the data may have broader interest and, collectively, may form the basis for image contrast exploration and simulations based on a wide range of dMRI analysis strategies.

## Background & Summary

Diffusion weighted MRI (dMRI) is highly sensitive to tissue microstructure, which makes it important as a tool in research and diagnostics. Traditional dMRI analysis relies on the diffusion tensor model^1,2^ where the diffusion signal is approximated with a Gaussian phase distribution. The microstructure of biological tissues, however, influences the diffusion process and causes the spin phase distribution to deviate from normal. This deviation is partially described by including the kurtosis term in the cumulant expansion^3^. The diffusion kurtosis imaging (DKI) framework^4^ captures this deviation and is seen as an indirect microstructural marker. DKI is an increasingly popular method to increase the sensitivity of dMRI to microstructure. In particular, the orientationally averaged kurtosis—the mean kurtosis (MK)—has been found to possess promising clinical potential. In an animal model of stroke, MK was found to improve the visualization of the ischemic lesion^5^ compared to mean diffusivity (MD) and to display different temporal dynamics than MD^6,7^. In human stroke, MK was also found to increase^8–11^. MK's potential value has also been reported in several other neurological applications: Parkinson’s disease^12^, epilepsy^13^, gliomas^14,15^, chronic mild stress^16^, attention deficit hyperactivity disorder (ADHD)^17^, traumatic brain injury^18^ and review in^19^, and normal development^20,21^.

Despite its potential, the exploration and application of DKI in everyday clinical imaging is held back by its large data requirement (causing long acquisition times) and computationally heavy postprocessing. In an effort to remove these limitations, strategies for fast kurtosis imaging have recently been proposed^22–24^. These strategies employ nine distinct diffusion encoding directions acquired at two different b-values to efficiently estimate the mean kurtosis using a definition based on the kurtosis tensor, W. In the same theoretical framework, the directional dependency of the kurtosis—the kurtosis fractional anisotropy (KFA)^25^—may be defined^22,26^ in a manner which is mathematically analogous to the fractional anisotropy (FA)^27^ known from diffusion tensor^2,28^ imaging (DTI). A compact scheme for KFA estimation by proxy was explored in a recent study^25^ where its contrast was also compared to conventional DTI and DKI contrasts. The data provided here allows users to perform both traditional DKI analysis and fast kurtosis analysis from data sets acquired in fixed rat brain and in human brain. One potential use of this data is for testing analysis software, postprocessing algorithms or b-value optimization, thus supplementing other publicly available data sets e.g. those presented in (refs 29,^30^) (data available at: cmic.cs.ucl.ac.uk/wmmchallenge/ and www.massive-data.org/). Furthermore, we provide high resolution dMRI data from rat spinal cord and a physical phantom which may be used to explore DKI contrasts and as a basis for simulations. The data was used in previously published analysis^22,25^. The data acquisition details are provided below. Details on data availability, formats, and organization are provided in the Data Records section and Table 1.

## Methods

All animal work was performed in accordance with relevant guidelines and regulations concerning animal experiments. All animal experimental protocols were approved by the Danish Animal Experiments Inspectorate (Dyreforsøgstilsynet). Human data acquisition was performed in accordance with the Declaration of Helsinki. All human experimental protocols were approved by the local ethics committee for research (De videnskabsetiske Komitéer for Region Midtjylland). Informed consent was obtained from all human subjects (one) prior to scanning. The data is made available raw, meaning that no pre-processing (smoothing, coregistration, spatial filtering, or normalization) was applied in any of the data sets. Throughout the method descriptions, SNR was calculated as the average signal in a homogenous region in the object imaged divided by the standard deviation of the signal in a background region, corrected for Rayleigh distribution in a standard manner^31^. Unless otherwise stated reported SNR levels were evaluated at b=0.

### MRI data obtained in fixed rat spinal cord

An adult male Wistar rat was euthanized and exsanguinated during intra-aortic perfusion fixation with isotonic saline containing heparin (10 IU ml^−1^), followed by 4% paraformaldehyde in phosphate-buffered saline (PBS) (pH 7.4). A section of spinal cord including the cervical enlargement was then dissected out and stored in 4% PFA for at least 6 weeks prior to imaging. The spinal cord segment was washed in PBS for 24 h prior to MR scanning to improve signal by removal of excess fixative. For imaging, the tissue was placed in a 5 mm NMR tube. Imaging was performed on a Bruker Biospec 16.4 T (Bruker Biospin, Germany) spectrometer equipped with microimaging gradients with a strength of 3 T/m. Data was acquired using a 5 mm saddle coil. DWI data acquisition was performed using a standard DW spin echo sequence. A total of 17 b-values equally distributed from 0-15 ms μm^−2^ were acquired. At each b-value, data was acquired along 9 gradient directions, so that the gradient directions at non-zero b-values in combination form a 144 point spherical design^32^. Imaging parameters were: TE=15.3 ms, TR=2500 ms, diffusion timings δ/Δ=2/8 ms, 3 averages. Acquisition time per b-value: 3 h 36 min. Twenty-five image slices were acquired at a resolution of 23 μm x 23 μm×120 μm, matrix size 192×192. Notice that an artifact caused by radio frequency feed-through (contamination) is present outside of the object. SNR is rather low in this data set (~7 at b=0) but the high spatial resolution of the data set in combination with the large range of b-values makes it applicable for DTI/DKI contrast exploration, and as a foundation for simulations based on DTI/DKI fits. The symmetry of the spinal cord also allows for averaging data across several slices to increase SNR. Down sampling and smoothing may extend the applicable b-value range even further. The raw, full data set is provided.

### MRI data obtained in fixed rat brain

This specimen was obtained using the same fixation protocol as above. After perfusion fixation the brain was removed and immersion fixed in fresh 4% paraformaldehyde solution for at least 6 weeks. Prior to imaging, the brain was washed in PBS for 24 h to improve signal by removal of excess fixative. Data was acquired using a Bruker Biospec 9.4 T (Bruker Biospin, Germany) MRI system equipped with a 15 mm quadrature coil. DWI data acquisition was performed using a standard DW spin echo sequence. A total of 15 b-values ranging from 0–3 ms μm^−2^ in steps of 0.2 ms μm^−2^ were acquired. At each b-value, data was acquired along 33 gradient directions. These directions were obtained by combination of a 3-dimensional 24-point spherical 7-design^32^ and the nine directions identified for fast estimation of mean kurtosis in ref. 22. Imaging parameters were: TE=23.3 ms, TR =4 s, diffusion timings δ/Δ=4/14 ms, 2 averages. Fifteen image slices were acquired at a resolution of 100 μm×100 μm x 500 μm, matrix size 128 × 128. SNR was approximately 75 at b=0 evaluated using the mean signal across all tissue.

### MRI data collected in a physical phantom

A physical phantom with fiber bundles equally distributed along the $\hat{x}$$\hat{y}$, and $\hat{z}$ directions was constructed. This phantom was used to mimic one imaging voxel with complex fiber distribution while at the same time allowing us to resolve each fiber direction separately. For this, a phantom was built using fresh asparagus stems. Stems were cut into 8 mm long sections and placed inside a cubic plastic container in a 3×3 design. The container was then filled with room temperature demineralized water and glass tools were used to remove air bubbles. Immediately after construction, the phantom was brought to the magnet. For scanning, the phantom was placed in an in-house built sample holder made from PE foam allowing the sample to be held tightly in place inside the MR coil. In this manner, sample movement (shaking) during acquisition was eliminated thereby avoiding image artifacts caused by bulk water motion. Imaging was performed on a horizontal 9.4 T Bruker Biospec system using a 40 mm quadrature coil. This coil is intended for mounting on an animal bed, but for these scans it was mounted in the magnet bore using a coil holder developed in-house. The scan protocol included an anatomical/structural scan and a DKI acquisition. The structural data was acquired with a FLASH sequence (TE=5.4 ms, TR=350 ms) in seven 1 mm thick slices with an in-plane resolution of 100 μm×100 μm, matrix size 280×280. Diffusion data was acquired using a standard diffusion weighted spin echo sequence. Data was recorded in the same seven slice planes as the structural data but with a lower in-plane resolution of 427 μm×427 μm, matrix size 64x64. Imaging parameters were TE=70.6 ms, TR=2700 ms, diffusion timings δ/Δ=6/60 ms, 4 averages. Fifteen encoding directions were obtained at b-values of 0, 0.5, 1.0, 1.8, 2.5, 3.5 ms μm^2^. The encoding directions were obtained from a 15 point spherical design^32^. The first slice is influenced by susceptibility effects near the water air surface. Therefore, we recommend to only analyze slices 2–7 as done in ref. 25.

### Human MRI data

Human data was acquired in one normal volunteer using a Siemens Trio 3 T equipped with a 32 channel head coil and a double spin echo DW EPI sequence. Motion of the subject's head during acquisition was avoided by padding inside the coil. DWI data was recorded at b=0 ms μm^−2^, and along 33 directions at b-values from 0.2–3 ms μm^−2^ in steps of 0.2 ms μm^−2^. The encoding scheme was constructed as a combination of a 24 point spherical design^32^ and the nine directions identified for rapid kurtosis estimation in ref. 22. CSF suppression (inversion recovery) was employed as recommended in ref. 33. Imaging parameters were TR=7200 ms, TE=116 ms, TI=2100 ms, 19 consecutive slices were acquired at isotropic resolution of 2.5 mm, matrix size 96×96, phase encoding direction A.-P. SNR~39 at b=0 evaluated using the mean signal across all tissue types.

Anatomical data is also provided. This data consists of a 1 mm isotropic T1 weighted 3D MPRAGE acquired in the sagittal orientation, matrix size 256 × 256 × 176. Scan parameters were: TE=3.7 ms, TR=2430 ms, Inversion time (TI)=960 ms, Flip angle=9°, 2 averages.

## Data Records

The MRI data acquired in fixed rat spinal cord is provided raw (no smoothing or registration has been performed). The data is available in Rat_spinal_cord.zip which contains dMRI data and corresponding b-values and gradient table (Data Citation 1). Details are provided in Table 1.

The raw MRI data acquired in fixed rat brain can be found in Rat_brain.zip which contains dMRI data and corresponding b-values and gradient table (Data Citation 1). Details are provided in Table 1.

The raw MRI data acquired in the physical phantom is bundled in Phantom_data.zip which contains dMRI data with corresponding b-values and gradient table, and a structural scan in the same slice positions (Data Citation 1). Details are provided in Table 1.

The raw MRI data acquired in human brain can be found in human_brain.zipt which contains dMRI data with corresponding b-values and gradient tables, and a structural scan (Data Citation 1). Details are provided in Table 1.

## Usage Notes

All data is provided as matlab files (.mat) and in the nifti format. The human data is also provided as dicom files. The DWI data is provided raw so no preprocessing has been applied to any of the data sets. This allows users to employ their preferred pre- and postprocessing combination and assess data quality e.g. drift effects, SNR etc. directly. The data is stored either as a 4D matrix with ordered with spatial dimensions first: x, y, slice, diffusion encoding. In these cases the diffusion encoding order corresponds to the accompanying vector of effective b-values (in ms μm^−2^) and gradient encoding directions (as normalized cartesian vectors). In case of a 5D data matrix the structure is x,y,slice,gradient encoding direction,b-value with the order of encoding directions and b-values given by the corresponding vectors. For Bruker data method files are available on request (contact corresponding author).

## Additional Information

**How to cite**: Hansen, B. & Jespersen S. N. Data for evaluation of fast kurtosis strategies, b-value optimization and exploration of diffusion MRI contrast. *Sci. Data* 3:160072 doi: 10.1038/sdata.2016.72 (2016).

## Supplementary Material



## Figures and Tables

**Table 1:** Overview of the data contained in Data Citation 1.

**Subjects**	**Protocol**	**Data**	**File name**
Fixed rat spinal cord	Diffusion MRI at 16.4 T	*Data is provided in nifti format with b-values and difusion gradient vectors in text format.* *The data is also provided in the matlab.mat file format:* ratSC.matThis matlab file contains raw dMRI data (ratSC_DWI_data), corresponding effective b-values (effective_b) and gradient table (DWI_encoding_directions), and masks for all slices (masks)	Rat_spinal_cord.zip
Fixed rat brain	Diffusion MRI at 9.4 T	*Data is provided in nifti format with b-values and difusion gradient vectors in text format.* *The data is also provided in the matlab.mat file:* rat_brain.matThis matlab file contains raw dMRI data (rat_brain_data), corresponding b-values (nominal_bvalues and effective_b, the latter includes b-value contributions from imaging gradients) and gradient table (DWI_encoding_directions_pr_b_value).	Rat_brain.zip
Physical phantom	Diffusion MRI at 9.4 TStructural scan	*Data is provided in nifti format with b-values and difusion gradient vectors in text format.* *The data is also provided in the matlab.mat file format:* phantom_data.matThis matlab file contains raw dMRI data (phantom_data), corresponding b-values (effective_b), gradient table (DWI_encoding_directions), and structural scan (structural_data).	Phantom_data.zip
Normal human brain1 volunteer	Diffusion MRI at 3 TT1 weighted anatomical data	*Diffusion MRI data is provided in both dicom and nifti format with b-values and difusion gradient vectors in text format.* *The structural T1 scan is also provided as dicom and nifti.* *The data set is also provided in the matlab.mat file:* human_brain.matThis matlab file contains raw dMRI data (human_brain_data), corresponding b-values (effective_b), and gradient table (DWI_encoding_directions). Anatomical data can be found in the variable 'T1_anat'.	Human_brain.zip
